# Insights into the Interaction of LVV-Hemorphin-7 with Angiotensin II Type 1 Receptor

**DOI:** 10.3390/ijms22010209

**Published:** 2020-12-28

**Authors:** Amanat Ali, Elizabeth K. M. Johnstone, Bincy Baby, Heng B. See, Angela Song, K. Johan Rosengren, Kevin D. G. Pfleger, Mohammed Akli Ayoub, Ranjit Vijayan

**Affiliations:** 1 Department of Biology, College of Science, United Arab Emirates University, Al Ain PO Box 15551, UAE; 201790370@uaeu.ac.ae (A.A.); bincy_baby@uaeu.ac.ae (B.B.); 2 Molecular Endocrinology and Pharmacology, Harry Perkins Institute of Medical Research, QEII Medical Centre, Nedlands, WA 6009, Australia; liz.johnstone@perkins.uwa.edu.au (E.K.M.J.); ethan.see@perkins.uwa.edu.au (H.B.S.); kevin.pfleger@perkins.uwa.edu.au (K.D.G.P.); 3 Centre for Medical Research, The University of Western Australia, Crawley, WA 6009, Australia; 4 Australian Research Council Centre for Personalised Therapeutics Technologies, Canberra, NSW 2609, Australia; 5 School of Biomedical Sciences, Faculty of Medicine, The University of Queensland, St Lucia, QLD 4072, Australia; a.song@uq.edu.au (A.S.); j.rosengren@uq.edu.au (K.J.R.); 6 Dimerix Limited, Nedlands, WA 6009, Australia; 7 Zayed Center for Health Sciences, United Arab Emirates University, Al Ain PO Box 15551, UAE

**Keywords:** LVV-hemorphin-7, AngII, AT1R, NanoBRET, molecular docking, molecular dynamics, PAM

## Abstract

Hemorphins are known for their role in the control of blood pressure. Recently, we revealed the positive modulation of the angiotensin II (AngII) type 1 receptor (AT1R) by LVV-hemorphin-7 (LVV-H7) in human embryonic kidney (HEK293) cells. Here, we examined the molecular binding behavior of LVV-H7 on AT1R and its effect on AngII binding using a nanoluciferase-based bioluminescence resonance energy transfer (NanoBRET) assay in HEK293FT cells, as well as molecular docking and molecular dynamics (MD) studies. Saturation and real-time kinetics supported the positive effect of LVV-H7 on the binding of AngII. While the competitive antagonist olmesartan competed with AngII binding, LVV-H7 slightly, but significantly, decreased AngII’s k_D_ by 2.6 fold with no effect on its B_max_. Molecular docking and MD simulations indicated that the binding of LVV-H7 in the intracellular region of AT1R allosterically potentiates AngII binding. LVV-H7 targets residues on intracellular loops 2 and 3 of AT1R, which are known binding sites of allosteric modulators in other GPCRs. Our data demonstrate the allosteric effect of LVV-H7 on AngII binding, which is consistent with the positive modulation of AT1R activity and signaling previously reported. This further supports the pharmacological targeting of AT1R by hemorphins, with implications in vascular and renal physiology.

## 1. Introduction

Hemorphins are endogenous hemoglobin-derived peptides implicated in numerous physiological and pathophysiological situations including spatial learning, inflammation, analgesia, and transient hypotension [1,2,3,4,5,6,7]. The molecular targets of hemorphins comprise the cell-surface G protein-coupled receptor (GPCR) members such as the opioid receptors [4,8,9,10,11] and also the membrane AngIV receptor [3,8]. Moreover, hemorphins are also known for their inhibitory effects on the angiotensin-converting enzyme (ACE), a key enzyme in the renin–angiotensin system (RAS). Inhibition of ACE leads to reduced angiotensin II (AngII) levels, which are associated with the well-known anti-hypertensive properties of hemorphins [6,12,13]. Recently, we described for the first time the AngII type 1 receptor (AT1R) as a second GPCR to be pharmacologically targeted by the decapeptide LVV-hemorphin-7 (LVV-H7) in vitro with a positive modulation of its activity and downstream signaling pathways in HEK293 cells [14]. These effects were observed on the canonical Gαq/inositol phosphate pathway of AT1R, as well as on the phosphorylation of the extracellular signal-regulated-kinases (ERK1/2) [14]. The positive modulation of AT1R implies a hypertensive effect and this was surprising and not consistent with the anti-hypertensive properties of hemorphins through the inhibition of ACE. This further indicates the complexity of the interplay between hemorphins and the physiological systems such as the vascular system.

In this study, we provide early insights into the binding of LVV-H7 to AT1R by using the NanoBRET technology recently developed for hormone-receptor binding in real-time and live cells [15] as well as molecular docking and long molecular dynamics (MD) simulations.

## 2. Results

### 2.1. LVV-H7 Positively Affected AngII Binding on AT1R in Live HEK293 Cells

**Figure 1:**
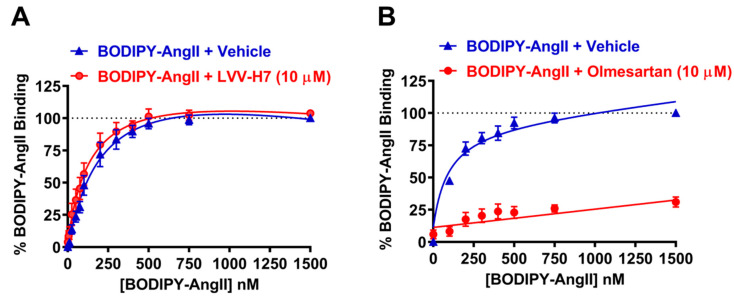
Saturation binding of BODIPY-angiotensin II (AngII) to AngII type 1 receptor (AT1R) using NanoBRET assay in live HEK293FT cells. HEK293FT cells transiently expressing Nluc-AT1R (NanoBRET donor) were first treated with 10 μM LVV-H7 or vehicle (**A**) or 1 μM olmesartan medoxomil or vehicle (**B**) for 20 min at 37 °C. Then, cells were incubated with increasing doses of BODIPY-AngII (NanoBRET acceptor) for 40 min at 37 °C before BRET signals were measured. Data are presented as % BODIPY-AngII binding by setting 100% as the maximal NanoBRET signal obtained with BODIPY-AngII in the absence of LVV-H7 (**A**) or olmesartan medoxomil (**B**). Data are mean ± SEM of four (**A**) or three (**B**) independent experiments performed in duplicate.

**Figure 2:**
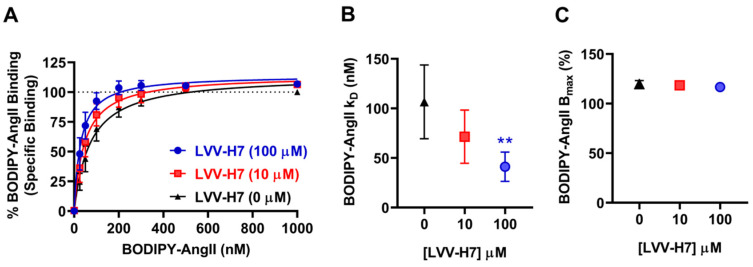
Specific binding of BODIPY-AngII to AT1R in the presence of LVV-H7 using NanoBRET assay in live HEK293FT cells. (**A**) HEK293FT cells transiently expressing Nluc-AT1R (NanoBRET donor) were first treated with 1 μM olmesartan medoxomil or vehicle and then immediately treated with 0, 10, or 100 μM LVV-H7. Following a 20-min incubation at 37 °C, cells were then treated with increasing doses of BODIPY-AngII (NanoBRET acceptor) for 40 min at 37 °C before BRET signals were measured. Specific binding curves were generated by subtracting olmesartan medoxomil-treated wells from vehicle-treated wells for each concentration of LVV-H7. Data are presented as % BODIPY-AngII binding by setting 100% as the maximal NanoBRET signals obtained with BODIPY-AngII in the absence of LVV-H7. (**B**) k_D_ and (**C**) B_max_ data from panel (**A**). The vehicle B_max_ value shown in panel (**C**) corresponds to the saturating value estimated by Prism analysis in panel (**A**) which is beyond the actual experimental B_max_ value. Data are mean ± SEM of six independent experiments performed in duplicate. ** *p*-value < 0.01.

We previously showed a positive allosteric modulation of AT1R activity and signaling by LVV-H7 in HEK293 cells [14]. In this study, we examined the effect of LVV-H7 on the binding of AngII on AT1R using NanoBRET technology in real-time and intact cells as previously reported [15]. First, we performed steady-state saturation binding experiments with BODIPY-labelled AngII (BODIPY-AngII) in the absence or presence of 10 μM of LVV-H7. As shown in Figure 1A, LVV-H7 did not compete for binding with BODIPY-AngII, in contrast to the ~75% displacement of binding observed when treated with the competitive AT1R antagonist olmesartan medoxomil (Figure 1B). As Figure 1A appeared to show a small increase in binding affinity of BODIPY-AngII in the presence of LVV-H7, we investigated this further by generating BODIPY-AngII-specific binding curves in the presence of increasing concentrations of LVV-H7 (Figure 2). Here we found that LVV-H7 significantly potentiated the binding of BODIPY-AngII (mean K_D_ ± SEM: 106.6 ± 37.1 nM at 0 μM LVV-H7 vs. 41.2 ± 14.7 nM at 100 μM LVV-H7; *n* = 6, *p* < 0.01 for paired ANOVA) (Figure 2A,B), without altering the maximum binding (Figure 2C). Such an effect suggests a positive allosteric interaction between LVV-H7 and BODIPY-AngII for binding to AT1R, which may explain its positive action on AT1R activity and signaling [14].

**Figure 3:**
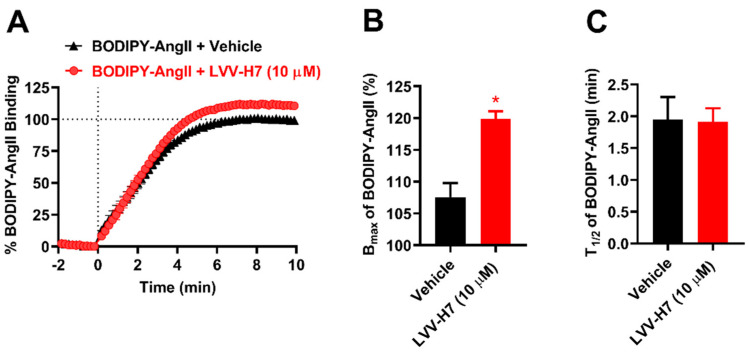
Real-time kinetics of the binding of BODIPY-AngII to AT1R in the presence of LVV-H7 using NanoBRET assay in live HEK293FT cells. (**A**) HEK293FT cells transiently expressing Nluc-AT1R (NanoBRET donor) were first treated with 10 μM LVV-H7 or vehicle for 30 min. BRET signals were then measured immediately after treatment of cells with 100 nM of BODIPY-AngII. (NanoBRET acceptor). Data are presented as % BODIPY-AngII binding by setting 100% as the maximal NanoBRET signals obtained with BODIPY-AngII in the absence of LVV-H7. (**B**) B_max_ and (**C**) T_1/2_ data from panel (A). Data are mean ± SEM of four independent experiments performed in duplicate. * *p*-value < 0.05.

To further explore this, we also carried out real-time binding kinetics using a near k_D_ dose of BODIPY-AngII (100 nM) in the absence or presence of 10 μM of LVV-H7. As shown in Figure 3, pre-incubation of cells with LVV-H7 significantly increased the maximal binding of BODIPY-AngII (mean B_max_ ± SEM = 108 ± 2% vs. 120 ± 1%; *n* = 4, *p* < 0.05 for paired t-test) with no effect on the binding rate (mean T_1/2_ ± SEM = 1.95 ± 0.18 min vs. 1.91 ± 0.11 min; *n* = 4, *p* > 0.05 for paired t-test). Overall, these in vitro NanoBRET data exclude any binding of LVV-H7 in the orthosteric binding site of AT1R suggesting the existence of an allosteric binding mode.

### 2.2. Molecular Docking and Molecular Dynamics Studies

#### 2.2.1. LVV-H7 Binds to an Intracellular Site of AT1R

**Figure 4:**
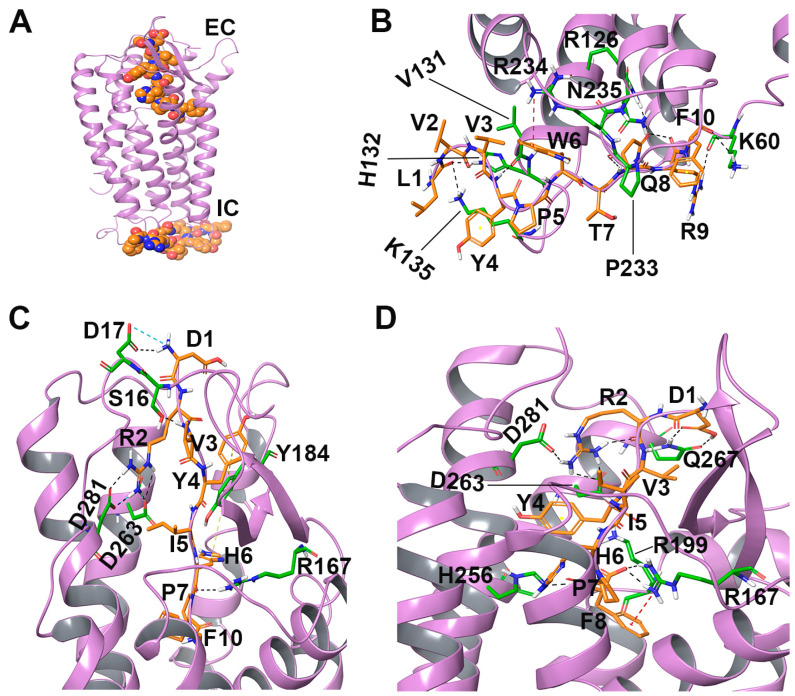
Structure of AT1R with the docked peptides. AT1R structure is shown in purple cartoon representation while its interacting residues are shown in green stick representation. Docked peptides are shown in orange stick representation. Black, yellow, and red dotted lines represent hydrogen bonds, π–π interactions, and π–cation interactions, respectively. (**A**) Structure of AT1R with AngII docked in the orthosteric site and LVV-H7 docked to the intracellular site shown in space-filling representation, (**B**) docked pose of LVV-H7 and the interactions it forms with AT1R, (**C**) docked pose of AngII in the absence of LVV-H7 and the interactions it forms with AT1R, and (**D**) docked pose of AngII in the presence of LVV-H7 and the interactions it forms with AT1R. For clarity, only important interactions are shown in (**D**). The complete list of interactions formed by LVV-H7 and AngII in the absence and presence of LVV-H7 is given in Table 1.

SiteMap analysis identified three potential binding sites in AT1R – two were intracellular and the third was the extracellular orthosteric site. Since the in vitro data indicated positive allosteric effects of LVV-H7, two potential binding cavities outside the orthosteric site were explored. LVV-H7 was docked to intracellular sites of AT1R identified by SiteMap. However, one of the intracellular sites produced extremely poor docking results and was therefore excluded from further studies. The best-docked pose of the LVV-H7-AT1R complex in the other site produced a GlideScore (GScore) docking score of −10.51 kcal/mol. Molecular mechanics-generalized Born surface area (MM-GBSA)-based binding free energy was estimated as −115.53 kcal/mol (Table 1). The docked conformation of LVV-H7 in this site interacted with intracellular loops 2 and 3, and the cytoplasmic end of transmembrane helices 3 and 6 (TM3 and TM6) of AT1R. The first three residues of the decapeptide formed hydrogen bonds with His132 and Lys135, a hydrophobic interaction with Tyr127, and an electrostatic interaction with Arg137 (Figure 4B and Supplementary Figure S1). The side chain of LVV-H7 Tyr4 formed a hydrogen bond with Val131 (Figure 4B). Pro5, Trp6, and Thr7 of LVV-H7 formed hydrophobic interactions with Ile130, Pro133, Met134, and Ala221 of AT1R. Trp6 also formed π-cation interaction with Arg234. Gln8 formed hydrogen bonds with Arg126 and Pro233, while, Arg9, and Phe10 formed hydrogen bonds with Lys60 and Asn235. The C-terminal residues Gln8, Arg9, and Phe10 of LVV-H7 also exhibited hydrophobic interactions with Val62, Ala129, Leu138, Met142, Ala225, and Ile238, electrostatic interactions with Lys230 and Lys232, and polar interactions with Thr61 (Table 1 and Supplementary Figure S1).

**Figure 5:**
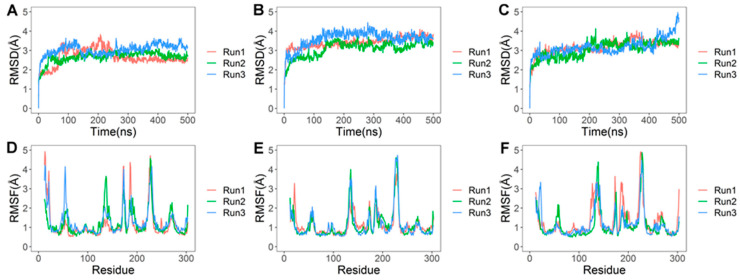
Plots of root mean square deviation (RMSD) and root mean square fluctuation (RMSF) of protein Cα atoms extracted from three independent 500 ns simulations of AT1R–peptide complexes. Data from three independent runs are plotted in blue, green, and red colors. (**A**) RMSD of AT1R-LVV-H7 complex, **(B**) RMSD of AT1R-AngII complex, (**C**) RMSD of AT1R-LVV-H7-AngII complex, (**D**) RMSF of AT1R-LVV-H7 complex, (**E**) RMSF of AT1R-AngII complex, and (**F**) RMSF of AT1R-LVV-H7-AngII complex.

**Figure 6:**
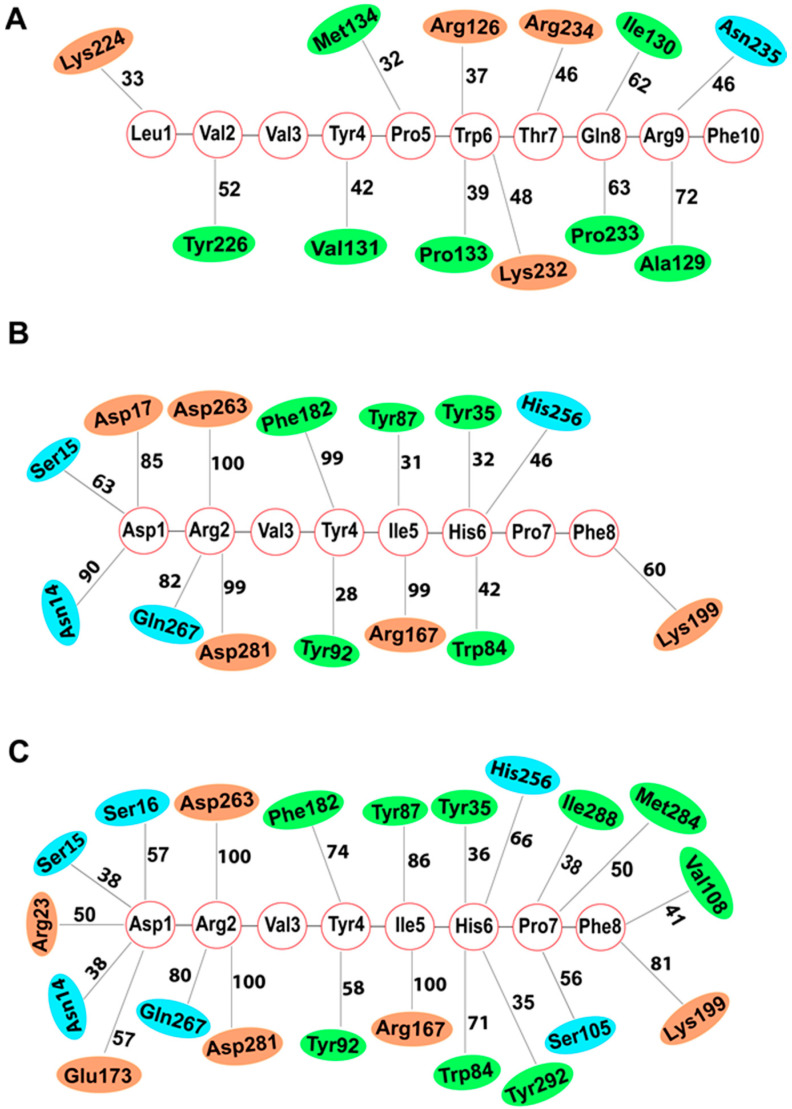
Average percentage of simulation time from three 500-ns runs during which AT1R residues maintained contact with LVV-H7 and AngII. The average percentage of simulation time is shown next to the line. Histograms showing the interaction contact time from each of the three simulations can be found in Supplementary Figure S2. Charged, hydrophobic, and polar residues of AT1R are represented with orange, green, and blue colors, respectively. (**A**) Average percentage of simulation time an AT1R residue maintained contact with LVV-H7, (**B**) average percentage of simulation time an AT1R residue maintained contact with AngII, and (**C**) average percentage of simulation time an AT1R residue maintained contact with AngII in the presence of LVV-H7.

Five hundred nanosecond all-atom MD simulations of the docked system embedded in a membrane were performed in triplicate to investigate the binding dynamics and stability of the AT1R-LVV-H7 complex using Desmond [16]. Protein structure in the simulated systems remained stable with a protein Cα root mean square deviation (RMSD) under 3.5 Å (Figure 5A). Residue level root mean square fluctuation (RMSF) highlighted that intracellular loops (ICL) ICL1, ICL2, and ICL3 and extracellular loop (ECL) ECL2 were more flexible while membrane-embedded regions exhibited limited fluctuations (Figure 5D). The decapeptide interacted consistently with ICL2 and ICL3, while its interactions with TM3 and TM6 were considerably weak. The N-terminal residues (Leu1 and Val2) formed intermittent interactions with Lys224 and Tyr226 of ICL3 (Figure 6A and Supplementary Figure S2A). Tyr4, and Pro5 formed intermittent interactions with Val131 and Met134 of ICL2, respectively. Trp6 of the peptide formed intermittent interactions with Arg126, Pro133, and Lys232 of TM3, ICL2, and ICL3 in all simulation runs while Thr7 formed consistent interactions with Arg234 during simulations. Gln8 exhibited sustained hydrophobic interactions with Ile130 and Pro233. Arg9 also produced sustained interaction with Ala129 of TM3 in all simulations (Figure 6A and Supplementary Figure S2A). These ICL2 residues have been reported to form an allosteric site in other GPCRs [17,18,19,20].

#### 2.2.2. Intracellular Binding of LVV-H7 to AT1R Potentiated the Binding of AngII

The effect of intracellular binding of LVV-H7 on AngII binding affinity was evaluated. AngII was first docked in the orthosteric site of AT1R. In the docked pose, the C-terminus of AngII was embedded deep in the active site and the N-terminus was extended towards the lid (Figure 4C). The best binding pose of Ang II had a GScore of −13.03 kcal/mol. The calculated MM-GBSA binding energy for this pose was −125.03 kcal/mol (Table 1). AngII formed multiple hydrogen bonds as well as hydrophobic, electrostatic and polar interactions that were consistent with previous studies (Supplementary Figure S3) [21,22]. Interactions between AngII and AT1R are provided in Table 1.

Next, the last frame from a simulation of LVV-H7 docked to AT1R intracellularly was taken and AngII was docked to the orthosteric binding site in this complex. Interestingly, the binding of AngII was improved. The best binding pose of AngII produced a GScore of −14.67 kcal/mol and MM-GBSA binding free energy of −142.81 kcal/mol (Table 1). This suggested that intracellular binding of LVV-H7 allosterically enhanced the binding affinity of AngII in the extracellular orthosteric binding site. This observation is consistent with the NanoBRET data obtained in HEK293FT cells (Figure 1, Figure 2 and Figure 3). Notably, Asp1, Tyr4, Pro7, and Phe8 residues of AngII were positioned differently when LVV-H7 was bound to AT1R (Figure 4C,D). The C-terminal of AngII bound slightly deeper inside the binding pocket in the presence of LVV-H7 (Figure 4C,D). This allowed Phe8 of AngII to form hydrogen bonds with Arg167, Lys199, His256, and Thr260. It also formed π–π stacking with His166 and Phe204. His6 of AngII formed a hydrogen bond with Arg167 in the absence of LVV-H7 (Figure 4C,D). Tyr4 and Ile5 of AngII formed hydrogen bonds with Tyr87, Phe182, and Val280 when LVV-H7 was bound (Figure 4D and Table 1). However, without LVV-H7, Tyr4 formed a π–π stacking with Tyr184 (Figure 4C). The sidechain amino groups of Arg2 of AngII formed hydrogen bonds with Asp263 and Asp281 of AT1R, with and without LVV-H7 bound. Asp1 and Val3 of AngII formed hydrogen bonds with Asp17 and Ser16, respectively, in the absence of LVV-H7. The backbone carbonyl and carboxyl groups of Asp1 formed a hydrogen bond with Gln267 in the presence of LVV-H7 (Figure 4C,D). Overall, AngII was able to form more hydrogen bonds, as well as hydrophobic and polar interactions, with AT1R when bound to LVV-H7 (Supplementary Figures S3 and S4).

The best binding poses of AT1R-AngII and AT1R-LVV-H7-AngII complexes were extracted, and the complexes were simulated for 500 ns in triplicate to assess the binding stability and dynamics of AngII with AT1R in the presence and absence of LVV-H7. The membrane-embedded AT1R structures with AngII and LVV-H7 bound remained stable throughout the duration of the simulations with a Cα root mean square deviation (RMSD) that was under 4 Å (Figure 5B,C). RMSF of AT1R, where both LVV-H7 and Ang II were bound, showed that ICL2 (residues 131–137) and ECL2 (residues 180–191) fluctuated slightly more than AT1R bound to AngII only (Figure 5E,F).

The stability of interactions between AT1R and the peptides were assessed from the simulations of the AT1R-AngII complex in the presence and absence of LVV-H7 (Figure 6B,C). AT1R residues Trp84, Val108, Leu112, Arg199, and Ile288, located at the bottom of the orthosteric binding site, interacted with Pro7 and Phe8 of AngII (Supplementary Figures S3 and S4). Arg199 was observed to be close to Phe8 of AngII in the AT1R-LVV-H7-AngII complex, and this inward movement was linked to a more sustained interaction of Arg199 with Phe8 during simulations when compared to the AT1R-AngII complex (Figure 6B,C). The interaction of Phe8 with Arg199 has been demonstrated to be an essential contact associated with AT1R activation [23,24]. Phe8 also formed consistent hydrophobic interactions with Val108 and intermittent interactions with Leu112 when LVV-H7 was bound. This interaction was observed in only one of the simulations when LVV-H7 was not bound to AT1R (Figure 6C and Supplementary Figure S2B,C). His6 showed consistent interactions with Trp84 of AT1R bound with LVV-H7 in all runs of simulation, while the same interaction was formed in only one run in the absence of LVV-H7. His6 of AngII also produced intermittent interactions with Tyr35 and His256. However, these interactions were better when LVV-H7 was bound to AT1R. Additionally, His6 also formed limited interactions with Tyr292 of AT1R in all simulations of AT1R-LVV-H7-AngII complex. This interaction was observed to be very weak when LVV-H7 was not bound to AT1R. Pro7 of AngII formed intermittent interactions with Ser105 and Ile288 residues, which were better when LVV-H7 was bound to AT1R. Ile5 maintained sustained interactions with Arg167 in both cases for the full duration of the simulations. Arg167 is an important residue for AT1R when compared to other structurally similar GPCRs and plays a vital role in defining ligand binding affinity [25]. Ile5 also formed consistent interactions with Tyr87 in all simulation runs when LVV-H7 was bound. This interaction was observed in only one of the simulations when LVV-H7 was not bound to AT1R. Tyr4 of AngII exhibited sustained interactions with Tyr92 of AT1R only when LVV-H7 was bound (Figure 6C). However, Tyr4 produced more sustained interactions with Phe182 when LVV-H7 was not bound to AT1R. AngII residue Arg2 interacted with Asp263, Gln267, and Asp281 of AT1R with and without LVV-H7 throughout the simulations. Such an interaction between AT1R and AngII has been reported previously [26,27,28]. Asp1 of AngII formed more sustained interactions with Asn14, Ser15, and Asp17 of AT1R in the absence of LVV-H7. Asp1 intermittently interacted with Ser16, Arg23, and Glu173 when LVV-H7 was bound to AT1R. Docking and simulation data indicated that Asp1 of AngII was firmly held by hydrogen bonds as well as hydrophobic and polar interactions with residues present around the lid of the orthosteric binding pocket of AT1R such as Asn14, Ser15, and Asp17 when LVV-H7 was not bound. This prevented the C-terminus of AngII from binding deep inside the orthosteric site. However, in the presence of LVV-H7, the N-terminal of AngII formed less-stable interactions with Asn14, Ser15, Asp16, and Arg23 of AT1R (Figure 6B,C). The N-terminal of AngII, in the presence of LVV-H7, bound slightly deeper in the binding pocket and exhibited fewer interactions with the residues located around the lid of the orthosteric binding pocket of AT1R. This permitted AngII to bind stably deep inside the orthosteric site. Overall, AngII formed more sustained interactions with important residues of AT1R which are essential for its activation in the presence of LVV-H7. Such subtle changes support the higher binding affinity of AngII in the presence of LVV-H7.

MM-GBSA-based free energy of binding (∆G_bind_) was estimated using frames extracted every 50 ns from the MD simulations. AT1R-AngII simulations in the presence of LVV-H7 exhibited ∆G_bind_ (−187.9 ± 15.3 kcal/mol, −170.4 ± 12.2 kcal/mol, and −175.4 ± 6.6 kcal/mol) that were higher than AT1R-AngII simulations without LVV-H7 (−151.2.0 ± 10.6 kcal/mol, −135.2 ± 7.6 kcal/mol, and −158.4 ± 5.2 kcal/mol). The notable improvement in ∆G_bind_ compared to the initially docked structure indicated that AngII remained stably bound in the simulations and has a higher binding affinity in the presence of LVV-H7.

## 3. Discussion

Hemorphins are endogenous bioactive peptides produced during proteolytic cleavage of hemoglobin. Several studies have already demonstrated their pharmacological properties in different physiological and pathological conditions [3,4,5,6,7,8]. We had previously reported the positive modulation of AT1R-mediated signaling by LVV-H7 in HEK293 cells [14]. Here, we used in vitro NanoBRET technology and in silico approaches to investigate the allosteric effect of LVV-H7 on AngII binding to AT1R. The data rule out any binding of LVV-H7 in the orthosteric binding site of AT1R, and more importantly indicate the allosteric potentiation of AngII binding affinity by LVV-H7 through binding to intracellular regions of AT1R.

Molecular docking and long MD simulations were performed to determine the most suitable binding pose of LVV-H7 and its effect on AngII binding to AT1R. Extensive molecular docking runs identified an intracellular location, previously linked to allosteric modulation in other GPCRs, as the most suitable binding site of LVV-H7 [29]. MD simulations revealed that LVV-H7 binding in this intracellular (IC) site induced conformational changes in the orthosteric binding pocket associated with AngII binding. Interestingly, the C-terminus of AngII was embedded deeper in its binding site and formed sustained interactions with Trp84, Ser105, Val108, Lys199, His256, Met284, Ile288, and Tyr292 of AT1R when compared to the structure without LVV-H7 (Figure 4C,D and Figure 6B,C).

Positive allosteric binding sites of GPCRs have been identified in the past [18,30]. However, recent studies have fully characterized the potential of positive allosteric modulators in multiple GPCRs using in vitro and in silico approaches [31,32,33]. Allosteric binding sites located near the extracellular (EC) face and IC face in different GPCRs have been documented [31,34]. Allosteric sites near the orthosteric site in GPCRs often block the entry of other ligands [19,31,35]. However, this would not be a problem for allosteric ligands that target the IC region. DETQ, an allosteric modulator of dopamine 1 receptor binds preferentially to ICL2 and significantly enhanced the potency of dopamine [19]. Interestingly, LVV-H7 binds to the IC face of AT1R and predominantly targets the ICL2 (Figure 4B and Figure 6A). LVV-H7 targets ICL2 residues in AT1R that are not conserved in dopamine 1 receptor, such as Lys135 and Ser136, as well as the conserved residues Ile130 and Pro133 (Figure 4B and Supplementary Figure S1). These residues are located near the binding site of DETQ on ICL2 of dopamine 1 receptor [19]. Moreover, recent studies have shown that AT1R-stabilizing nanobodies target the intracellular side of AT1R, especially ICL2 residues, and enhance the binding affinity of AngII for AT1R [20]. This phenomenon shows complementary cooperativity anticipated for an allosteric interaction. Interestingly, LVV-H7 binds to these ICL2 residues of AT1R and potentiates the binding affinity (Figure 4 and Figure 6) and potency [14] of AngII. The allosteric binding of LVV-H7 in the intracellular regions of AT1R is consistent with the allosteric modulation of GPCRs through the targeting of their intracellular surfaces, which has been well documented with the lipidated peptides known as pepducins and even small molecules [26,27,29,36].

Evaluating the binding mode of AngII in the presence and absence of LVV-H7 revealed that AngII bound deeper in the orthosteric binding pocket in the presence of LVV-H7 (Figure 4B,C). The interaction between Phe8 of AngII with Lys199, located at the base of the orthosteric pocket of AT1R was improved in the presence of LVV-H7 (Figure 6B,C). Lys199 has been identified as a residue critical for AT1R activation [25,37]. Tyr35 and Trp84 are conserved in other GPCRs, such as chemokine and opioid receptors, and mutating these residues would abolish the binding of both peptide and synthetic ligands of AT1R [25,28,38]. In simulations, Tyr35 and Trp84 were observed to form more sustained interactions with AngII in the presence of LVV-H7 (Figure 6B,C). The AT1R activation cascade has also been shown to be initiated by van der Waals interaction between Phe8 and Ile288 [21]. Interestingly, intracellular binding of LVV-H7 enhanced the stability of the interaction between Phe8 and Ile288 (Figure 6B,C). This could lead to a more robust disruption of the Asn111–Asn295 interaction, and better AngII binding affinity and potency [21]. The existence of a stable hydrogen bond between Asn111 and Asn295 is characteristic of the inactive state of AT1R and its disruption is vital for AT1R activation [25,37]. Mutagenesis studies have also reported that Tyr87, Arg167, and Tyr292 are important for AngII binding [21]. Interestingly, these residues formed more sustained interactions with AngII in the presence of LVV-H7 (Figure 6B,C).

DRY, Yx7K, NPxxY, and helix-8 motifs are highly conserved among GPCRs and are located in the cytoplasmic region. These motifs, initially reported in rhodopsin, act as microswitches [39]. Functional studies of AT1R have illustrated the stability of these motifs in the inactive state [21,39]. The ICL2 residues of AT1R, where LVV-H7 binds, are associated with structural changes that induce a transition from an inactive to an active conformation in GPCRs [19]. Trajectory analysis of AT1R-LVV-H7 complex indicated that the binding of LVV-H7 to ICL2 and ICL3 of AT1R induced conformational changes in the DRY motif present at the cytoplasmic end of TM3 that is important for GPCR activation [19,21]. GPCR activation involves the breakage of a stable interaction that exists between the side chains of Asp125 and Arg126 of the DRF motif in the inactive state [21,40]. Interestingly, LVV-H7 binds to the DRF motif and forms an intermittent interaction with the Arg126 residue of AT1R throughout the simulations (Figure 4B and Figure 6A and Supplementary Figure S2A). Additionally, the sidechain of Arg126 was observed to slightly orient itself towards TM6 in the presence of LVV-H7. These changes have been associated with the opening of the groove where G-protein binds and could, therefore, enhance the coupling of G-protein to AT1R [21,41].

The concentration of hemorphins and the conditions of their release from hemoglobin under both physiological and pathophysiological conditions are not fully known. A few studies have reported the concentration of some forms of hemorphins in the blood. For instance, the serum VV-H7 level was reported to be around 5 µM in healthy individuals and this decreased to around 2 µM in the case of obese individuals [42]. The serum level of hemorphin-7 was reported to be 2.27 ± 0.63 μM in breast cancer patients when compared to healthy subjects (4.09 ± 1.05 μM) [43]. However, in another study, the baseline plasma level of hemorphin-7 was found to range between 0.2 and 6.9 nM [44]. Nonetheless, it is worth mentioning that LVV-H7 is the longest and most stable form normally generated before the hemorphin peptides. LVV-H7 also acts as a precursor for shorter hemorphins [4]. Therefore, it is likely that LVV-H7 level could be higher than other shorter derivatives of hemorphins.

In conclusion, this is the first study reporting the allosteric modulation of AT1R by LVV-H7. Additionally, this study identifies the intracellular domain as the most appropriate binding site for LVV-H7 and describes the mode of interaction between AT1R and LVV-H7. BRET data also indicate that LVV-H7 binds non-competitively. LVV-H7 allosterically potentiates the affinity and potency of AngII. These findings could help design novel allosteric modulators of AT1R to target the intracellular binding site.

## 4. Materials and Methods

### 4.1. cDNA Constructs and Ligands

The NanoLuc-AT1R coding plasmid (Promega, Madison, WI, USA) was used for its transient expression in HEK293FT cells as previously reported [15]. Olmesartan medoxomil (Sigma Aldrich, Castle Hill, NSW, Australia), and LVV-H7 (LVVYPWTQRF)(New England Peptide, Gardner, MA, USA) were used as ligands. 

To generate BODIPY-AngII the AngII sequence was assembled on 2-chlorotrityl resin (0.8 mmol/g loading) using standard Fmoc chemistry with 2-(1H-benzotriazol-1-yl)-1,1,3,3-tetramethyluronium hexafluorophosphate (HBTU)/*N*,*N*-diisopropylethylamine (DIPEA) activation. Briefly, 2-chlorotrityl resin was swelled in dichloromethane (DCM) for 1 h, then 2 equiv. of Fmoc-Phe-OH with 8 equiv. of DIPEA were added to the resin and allowed to react for 30 min. Unreacted sites were blocked with DCM/MeOH/DIPEA (17:2:1). The N-terminal Fmoc protection group was removed with 20% piperidine in dimethylformamide (DMF, 2 × 5 min). All remaining couplings were performed on a CS Bio CS336X automated synthesizer with 4 equiv. of Fmoc-amino acid, 8 equiv. of HBTU, and 8 equiv. of DIPEA. The peptide was cleaved from the resin with trifluoroacetic acid (TFA)/triisopropylsilane (TIPS)/Milli Q (95:2.5:2.5) and lyophilized. The crude peptide was dissolved in 10% acetonitrile and purified by RP-HPLC on a Prominence HPLC system (Shimadzu) using a semi-preparative Grace Vydac C18 column (250 mm × 10 mm, 10 µm) at a 1% gradient and a flow rate of 3 mL/min. Electrospray mass spectrometry (API2000, AB Sciex) was used to confirm the molecular weight. For N-terminal labelling the pure lyophilized peptide was dissolved in DMF at 1 mM concentration with 100 mM trimethylamine, and 1 mM of BODIPY^TM^ 630/650-x NHS ester (ThermoFisher, Brisbane, QLD, Australia), and stirred for 24 h, protected from light. The reaction was monitored with analytical RP-HPLC and electrospray mass spectrometry.

### 4.2. Cell Culture and Transfection

Human embryonic kidney (HEK) 293FT cells (Thermo Fisher Scientific) were cultured and maintained at 37 °C, 5% CO_2_ in complete medium (Dulbecco’s modified Eagle’s medium (DMEM) containing 0.3 mg/mL glutamine, 100 IU/mL penicillin, and 100 μg/mL streptomycin) supplemented with 10% fetal calf serum (FCS) (Bovogen Biologicals, Victoria, Australia) and 400 μg/mL Geneticin (Thermo Fisher Scientific). Transient transfections were carried out 24 h after seeding 600,000 cells/well of a 6-well plate. FuGENE 6 (Promega) transfection reagent was used according to the manufacturer’s instructions. Cells were harvested with 0.05% Trypsin-EDTA 24 h after transfection and seeded into poly-L-lysine (Sigma Aldrich) coated white 96-well plates (Greiner Bio-One, Frickenhausen, Germany) at 80,000 cells/well in phenol red-free DMEM containing 25 mM HEPES, 0.3 mg/mL glutamine, 100 IU/mL penicillin, and 100 μg/mL streptomycin supplemented with 5% FCS.

### 4.3. Saturation NanoBRET Assay

Cells were first treated with olmesartan medoxomil, LVV-H7 and/or vehicle (as described in figure legends) for 20 min at 37 °C followed by addition of increasing doses of BODIPY-AngII and incubation for a further 40 min at 37 °C. The NanoLuc substrate furimazine (Promega) was then added to a final concentration of 10 μM and light emission was measured at 37 °C using the filters 450 nm (80-nm bandpass) and >610 nm (longpass) on the LUMIstar or CLARIOstar multilabel plate reader (BMG Labtech (Australia), Mornington, VIC, Australia).

### 4.4. Real-Time NanoBRET Kinetics

Cells were first treated with 10 μM of LVV-H7 for 30 min at 37 °C. Then, the Nanoluc substrate furimazine (10 μM) was added and BRET signals were immediately measured at 37 °C before and after adding 100 nM of BODIPY-AngII using the CLARIOstar multilabel plate reader (BMG Labtech (Australia)) as described above.

### 4.5. Molecular Docking

Three-dimensional structure of AT1R was downloaded from the Protein Data Bank (PDB) with PDB ID 4ZUD [25]. The Protein Preparation Wizard of Schrödinger Suite 2016-4 (Schrödinger Release 2016-4) was used to prepare the structure of AT1R for in silico studies. Protein structure was preprocessed to remove water molecules, metal ions, and cofactors as well as assign bond orders, adjust ionization states, and to fix mis-oriented groups. Standard protonation states at pH 7 were used for protein residues after the incorporation of hydrogen atoms. Geometrically stable structure was obtained by optimizing and minimizing the preprocessed structure [45]. Schrödinger SiteMap was used to identify potential binding sites (Schrödinger 2016-4: SiteMap). Receptor grids were generated encompassing the identified sites with the default parameters for van der Waals scaling factor (1.00) and charge cutoff (0.25) by using the Optimized Potential for Liquid Simulations (OPLS) force field. A cubic search box was defined centered on the centroid of the binding site residues. Next, Schrödinger Glide’s standard precision (SP) method was used to perform flexible docking of the peptide in the identified sites with penalties applied for cis amide bonds [46]. LVV-H7 and AngII were docked to the processed AT1R structure, using Schrödinger Glide, to identify the best binding pose and to assess the related energetics [46]. The ConfGen algorithm was used to obtain multiple random conformations of LVV-H7 and AngII [47]. The docked poses were further optimized and minimized. The best-docked pose with lowest GlideScore value was recorded for each peptide [42]. The MM-GBSA method was used to compute the binding free energy of the docked poses by employing the VSGB 2.0 implicit solvent model [48] in Schrödinger Prime (Schrödinger Release 2016-4: Prime).

### 4.6. Molecular Dynamics Simulations

Five hundred nanosecond MD simulations were run in triplicate to evaluate the stability and dynamics of the best docked pose of each complex using Desmond [16]. The simulation system was described by the OPLS 2005 force field [49]. Three docked systems were simulated—LVV-H7 docked to AT1R, AngII docked to AT1R, and both LVV-7 and AngII docked to AT1R. The 500-ns simulations were run in triplicate with a different set of initial velocities. The AT1R complexes were embedded in a DPPC membrane and surrounded by single point charge water molecules [50,51]. A buffer space of 10 Å was maintained for each system and simulated with periodic boundary conditions. The system was neutralized and a 150 mM NaCl salt concentration was maintained by adding adequate numbers of ions and counterions. The simulation systems were minimized before performing production MD simulations using Desmond [16].

Each system was initially relaxed by following Desmond’s default relaxation protocol for membrane protein [48]. The relaxation protocol included minimization with and without restraints, simulation with gradually increasing temperature from 0 K to 300 K, gradual constraining and water barrier, simulation in an NPT ensemble in the presence of a water barrier with heavy atoms constraints, solvent and lipids equilibration in NPT, simulation in an NPT ensemble with the protein heavy atom constraint decreased from 10.0 to 2.0 kcal/mol, equilibration in an NPT ensemble in the presence of Cα atoms constrained at 2 kcal/mol, and finally a short simulation under NPT ensemble for 1.5 ns without any constraints. After the relaxation, 500-ns production simulations were performed with no restraints for each complex. A Nose–Hoover thermostat and isotropic Martyna–Tobias–Klein barostat were used to maintain the temperature at 300 K and pressure at 1 atm [52,53]. Coulombic interactions were evaluated with a cut-off of 9 Å with the short-range method. A time-reversible reference system propagator algorithm (RESPA) integrator was used with an inner time step of 2.0 fs and an outer time step of 6.0 fs [54]. Simulated data was saved at 100 ps intervals to simulation trajectories. At the end of each simulation, RMSD, RMSF, and protein–ligand interactions were computed from the trajectories.

### 4.7. Data Presentation and Statistical Analysis

The NanoBRET ratios were determined by dividing the >610 nm (longpass) emission by 450 nm (80-nm bandpass) emission and subtracting the background ratio. Then, the percentage of NanoBRET signals were calculated as described in figure legends. The saturation and kinetic curves were fitted with the appropriate nonlinear regression equations using GraphPad Prism software (San Diego, CA, USA). Statistical analyses were performed with paired one-way ANOVA or paired t-tests as appropriate to determine statistical significance between the different conditions. **** *p*-value < 0.0001, *** *p*-value < 0.001, ** *p*-value < 0.01, * *p*-value < 0.05, and ns *p*-value > 0.05.

## Figures and Tables

**Table 1:** AT1R residues that interacted with the best docked pose of LVV-H7 and AngII.

Peptide	AT1R PBD ID	GlideScore Docking Score (kcal/mol)	MM-GBSA Binding Energy (kcal/mol)	Residues Forming Hydrogen Bonds	Residues Forming Hydrophobic Interactions	Residues Forming π-π Stacking or Cation-π Interactions
LVV-H7	4ZUD	−10.51	−115.53	Lys60, Arg126, Val131, His132, Lys135, Pro233, Asn235	Val62, Ala129, Ile130, Pro133, Met134, Leu138, Met142, Leu217, Ala221, Ala225, Ile238	Arg234
AngII	4ZUD	−13.03	−125.03	Ser16, Asp17, Arg167, Asp263, Asp281	Leu13, Cys18, Pro19, Ala21, Ile31, Tyr35, Trp84, Tyr87, Tyr92, Val108, Ile172, Ala181, Phe182, Trp253, Ile266, Ala277, Val280, Met284, Pro285, ILe288, Tyr292	Tyr184
AngII with LVV-H7	4ZUD	−14.67	−142.81	Tyr87, Arg167, Phe182, Lys199, His256, Asp263, Gln267, Val280, Asp281	Ile12, Leu13, Tyr35, Trp84, Tyr87, Tyr92, Val108, Ala106, Leu112, Ala163, Val179, Cys180, Ala181, Tyr184, Leu202, Trp253, Phe259, Ile266, Ala277, Ala283, Met284, Pro285, Ile288, Tyr292	His166, Phe204

## Supplementary Materials

The following are available online at https://www.mdpi.com/1422-0067/22/1/209/s1.

## References

[1] Hughes J., Smith T.W., Kosterlitz H.W., Fothergill L.A., Morgan B.A., Morris H.R. (1975). Identification of two related pentapeptides from the brain with potent opiate agonist activity. Nature.

[2] Moisan S., Harvey N., Beaudry G., Forzani P., Burhop K.E., Drapeau G., Rioux F. (1998). Structural requirements and mechanism of the pressor activity of Leu-Val-Val-hemorphin-7, a fragment of hemoglobin beta-chain in rats. Peptides.

[3] Albiston A.L., Pederson E.S., Burns P., Purcell B., Wright J.W., Harding J.W., Mendelsohn F.A., Weisinger R.S., Chai S.Y. (2004). Attenuation of scopolamine-induced learning deficits by LVV-hemorphin-7 in rats in the passive avoidance and water maze paradigms. Behav. Brain Res..

[4] Ali A., Alzeyoudi S.A.R., Almutawa S.A., Alnajjar A.N., Vijayan R. (2020). Molecular basis of the therapeutic properties of hemorphins. Pharmacol. Res..

[5] Cejka J., Zelezna B., Velek J., Zicha J., Kunes J. (2004). LVV-hemorphin-7 lowers blood pressure in spontaneously hypertensive rats: Radiotelemetry study. Pharmacol. Res..

[6] Ali A., Alzeyoudi S.A.R., Almutawa S.A., Alnajjar A.N., Al Dhaheri Y., Vijayan R. (2020). Camel Hemorphins Exhibit a More Potent Angiotensin-I Converting Enzyme Inhibitory Activity than Other Mammalian Hemorphins: An In Silico and In Vitro Study. Biomolecules.

[7] Cheng B.C., Tao P.L., Cheng Y.Y., Huang E.Y. (2012). LVV-hemorphin 7 and angiotensin IV in correlation with antinociception and anti-thermal hyperalgesia in rats. Peptides.

[8] Ali A., Baby B., Soman S.S., Vijayan R. (2019). Molecular insights into the interaction of hemorphin and its targets. Sci. Rep..

[9] Davis T.P., Gillespie T.J., Porreca F. (1989). Peptide fragments derived from the beta-chain of hemoglobin (hemorphins) are centrally active in vivo. Peptides.

[10] Liebmann C., Schrader U., Brantl V. (1989). Opioid receptor affinities of the blood-derived tetrapeptides hemorphin and cytochrophin. Eur. J. Pharmacol..

[11] Szikra J., Benyhe S., Orosz G., Darula Z., Piot J.M., Fruitier I., Monory K., Hanoune J., Borsodi A. (2001). Radioligand binding properties of VV-hemorphin 7, an atypical opioid peptide. Biochem. Biophys. Res. Commun..

[12] Zhao Q., Piot J.M. (1997). Investigation of inhibition angiotensin-converting enzyme (ACE) activity and opioid activity of two hemorphins, LVV-hemorphin-5 and VV-hemorphin-5, isolated from a defined peptic hydrolysate of bovine hemoglobin. Neuropeptides.

[13] Fruitier-Arnaudin I., Cohen M., Bordenave S., Sannier F., Piot J.M. (2002). Comparative effects of angiotensin IV and two hemorphins on angiotensin-converting enzyme activity. Peptides.

[14] Ali A., Palakkott A., Ashraf A., Al Zamel I., Baby B., Vijayan R., Ayoub M.A. (2019). Positive modulation of angiotensin II type 1 receptor–mediated signaling by LVV–hemorphin-7. Front. Pharmacol..

[15] Stoddart L.A., Johnstone E.K.M., Wheal A.J., Goulding J., Robers M.B., Machleidt T., Wood K.V., Hill S.J., Pfleger K.D.G. (2015). Application of BRET to monitor ligand binding to GPCRs. Nat. Methods.

[16] Bowers K.J., Chow D.E., Xu H., Dror R.O., Eastwood M.P., Gregersen B.A., Klepeis J.L., Kolossvary I., Moraes M.A., Sacerdoti F.D. Scalable algorithms for molecular dynamics simulations on commodity clusters. Proceedings of the SC’06 2006 ACM/IEEE Conference on Supercomputing.

[17] Zhang H., Qiao A., Yang D., Yang L., Dai A., de Graaf C., Reedtz-Runge S., Dharmarajan V., Han G.W., Grant T.D. (2017). Structure of the full-length glucagon class B G-protein-coupled receptor. Nature.

[18] Luderman K.D., Conroy J.L., Free R.B., Southall N., Ferrer M., Sanchez-Soto M., Moritz A.E., Willette B.K., Fyfe T.J., Jain P. (2018). Identification of positive allosteric modulators of the D1 dopamine receptor that act at diverse binding sites. Mol. Pharmacol..

[19] Wang X., Heinz B.A., Qian Y.W., Carter J.H., Gadski R.A., Beavers L.S., Little S.P., Yang C.R., Beck J.P., Hao J. (2018). Intracellular Binding Site for a Positive Allosteric Modulator of the Dopamine D1 Receptor. Mol. Pharmacol..

[20] Wingler L.M., McMahon C., Staus D.P., Lefkowitz R.J., Kruse A.C. (2019). Distinctive Activation Mechanism for Angiotensin Receptor Revealed by a Synthetic Nanobody. Cell.

[21] Singh K.D., Unal H., Desnoyer R., Karnik S.S. (2019). Mechanism of Hormone Peptide Activation of a GPCR: Angiotensin II Activated State of AT1R Initiated by van der Waals Attraction. J. Chem. Inf. Model..

[22] Fillion D., Cabana J., Guillemette G., Leduc R., Lavigne P., Escher E. (2013). Structure of the human angiotensin II type 1 (AT1) receptor bound to angiotensin II from multiple chemoselective photoprobe contacts reveals a unique peptide binding mode. J. Biol. Chem..

[23] Fillion D., Lemieux G., Basambombo L.L., Lavigne P., Guillemette G., Leduc R., Escher E. (2010). The amino-terminus of angiotensin II contacts several ectodomains of the angiotensin II receptor AT1. J. Med. Chem..

[24] Noda K., Saad Y., Karnik S.S. (1995). Interaction of Phe8 of angiotensin II with Lys199 and His256 of AT1 receptor in agonist activation. J. Biol. Chem..

[25] Zhang H., Unal H., Desnoyer R., Han G.W., Patel N., Katritch V., Karnik S.S., Cherezov V., Stevens R.C. (2015). Structural basis for ligand recognition and functional selectivity at angiotensin receptor. J. Biol. Chem..

[26] Zhang P., Leger A.J., Baleja J.D., Rana R., Corlin T., Nguyen N., Koukos G., Bohm A., Covic L., Kuliopulos A. (2015). Allosteric Activation of a G Protein-coupled Receptor with Cell-penetrating Receptor Mimetics. J. Biol. Chem..

[27] O’Callaghan K., Kuliopulos A., Covic L. (2012). Turning receptors on and off with intracellular pepducins: New insights into G-protein-coupled receptor drug development. J. Biol. Chem..

[28] Tan Q., Zhu Y., Li J., Chen Z., Han G.W., Kufareva I., Li T., Ma L., Fenalti G., Li J. (2013). Structure of the CCR5 chemokine receptor–HIV entry inhibitor maraviroc complex. Science.

[29] Covic L., Gresser A.L., Talavera J., Swift S., Kuliopulos A. (2002). Activation and inhibition of G protein-coupled receptors by cell-penetrating membrane-tethered peptides. Proc. Natl. Acad. Sci. USA.

[30] Bruns R.F., Fergus J.H. (1990). Allosteric enhancement of adenosine A1 receptor binding and function by 2-amino-3-benzoylthiophenes. Mol. Pharmacol..

[31] Kruse A.C., Ring A.M., Manglik A., Hu J., Hu K., Eitel K., Hubner H., Pardon E., Valant C., Sexton P.M. (2013). Activation and allosteric modulation of a muscarinic acetylcholine receptor. Nature.

[32] Congreve M., Oswald C., Marshall F.H. (2017). Applying Structure-Based Drug Design Approaches to Allosteric Modulators of GPCRs. Trends Pharmacol. Sci..

[33] De Amici M., Dallanoce C., Holzgrabe U., Trankle C., Mohr K. (2010). Allosteric ligands for G protein-coupled receptors: A novel strategy with attractive therapeutic opportunities. Med. Res. Rev..

[34] Bock A., Merten N., Schrage R., Dallanoce C., Batz J., Klockner J., Schmitz J., Matera C., Simon K., Kebig A. (2012). The allosteric vestibule of a seven transmembrane helical receptor controls G-protein coupling. Nat. Commun..

[35] Proska J., Tucek S. (1994). Mechanisms of steric and cooperative actions of alcuronium on cardiac muscarinic acetylcholine receptors. Mol. Pharmacol..

[36] Carlson K.E., McMurry T.J., Hunt III S.W. (2012). Pepducins: Lipopeptide allosteric modulators of GPCR signaling. Drug Discov. Today Technol..

[37] Zhang H., Unal H., Gati C., Han G.W., Liu W., Zatsepin N.A., James D., Wang D., Nelson G., Weierstall U. (2015). Structure of the Angiotensin receptor revealed by serial femtosecond crystallography. Cell.

[38] Wu B., Chien E.Y., Mol C.D., Fenalti G., Liu W., Katritch V., Abagyan R., Brooun A., Wells P., Bi F.C. (2010). Structures of the CXCR4 chemokine GPCR with small-molecule and cyclic peptide antagonists. Science.

[39] Elgeti M., Roman K., Martin H., Takefumi M., Eglof R., Patrick S., Oliver P., Friedrich S., Klaus P., Franz J. (2011). Conserved Tyr223(5.58) plays different roles in the activation and G-protein interaction of rhodopsin. J. Am. Chem. Soc..

[40] Matsoukas M.-T., Potamitis C., Plotas P., Androutsou M.-E., Agelis G., Matsoukas J., Zoumpoulakis P. (2013). Insights into AT1 receptor activation through AngII binding studies. J. Chem. Inf. Model..

[41] Rasmussen S.G., DeVree B.T., Zou Y., Kruse A.C., Chung K.Y., Kobilka T.S., Thian F.S., Chae P.S., Pardon E., Calinski D. (2011). Crystal structure of the β 2 adrenergic receptor–Gs protein complex. Nature.

[42] Maraninchi M., Feron D., Fruitier-Arnaudin I., Bégu-Le Corroller A., Nogueira P., Mancini J., Valéro R., Piot J.M., Vialettes B. (2013). Serum hemorphin-7 levels are decreased in obesity. Obesity.

[43] Cohen M., Ingrid Fruitier-Arnaudin S., Daniel B., Jean-Marie P. (2002). Serum levels of Hemorphin-7 peptides in patients with breast cancer. Clin. Chim. Acta.

[44] Glämsta L., Mørkrid L., Lantz I., Nyberg F. (1993). Concomitant increase in blood plasma levels of immunoreactive hemorphin-7 and beta-endorphin following long distance running. Regul. Pept..

[45] Sastry G.M., Adzhigirey M., Day T., Annabhimoju R., Sherman W. (2013). Protein and ligand preparation: Parameters, protocols, and influence on virtual screening enrichments. J. Comput. Aided Mol. Des..

[46] Friesner R.A., Banks J.L., Murphy R.B., Halgren T.A., Klicic J.J., Mainz D.T., Repasky M.P., Knoll E.H., Shelley M., Perry J.K. (2004). Glide: A new approach for rapid, accurate docking and scoring. 1. Method and assessment of docking accuracy. J. Med. Chem..

[47] Watts K.S., Dalal P., Murphy R.B., Sherman W., Friesner R.A., Shelley J.C. (2010). ConfGen: A conformational search method for efficient generation of bioactive conformers. J. Chem. Inf. Model..

[48] Li J., Abel R., Zhu K., Cao Y., Zhao S., Friesner R.A. (2011). The VSGB 2.0 model: A next generation energy model for high resolution protein structure modeling. Proteins.

[49] Shivakumar D., Williams J., Wu Y., Damm W., Shelley J., Sherman W. (2010). Prediction of absolute solvation free energies using molecular dynamics free energy perturbation and the OPLS force field. J. Chem. Theory Comput..

[50] Mark P., Nilsson L. (2001). Structure and dynamics of the TIP3P, SPC, and SPC/E water models at 298 K. J. Phys. Chem. A.

[51] Matyszewska D., Bilewicz R. (2008). DPPC monolayers as simple models of biological membranes for studies of interactions with perfluorinated compounds. Ann. UMCS Chem..

[52] Martyna G.J., Klein M.L., Tuckerman M. (1992). Nosé–Hoover chains: The canonical ensemble via continuous dynamics. J. Chem. Phys..

[53] Martyna G.J., Tobias D.J., Klein M.L. (1994). Constant pressure molecular dynamics algorithms. J. Chem. Phys..

[54] Tuckerman M., Berne B.J., Martyna G.J. (1992). Reversible multiple time scale molecular dynamics. J. Chem. Phys..

