# Supplementary material for: Insights into the Interaction of LVV-Hemorphin-7 with Angiotensin II Type 1 Receptor
Source: Int J Mol Sci. 2020 Dec 28;22(1):209. doi: 10.3390/ijms22010209 (PMC7795518; doi:10.3390/ijms22010209)
Supplement: Supplementary file 1 [file ijms-22-00209-s001.pdf]

Insights into the interaction of LVV-hemorphin-7 with Angiotensin II Type 1 Receptor

Amanat Ali[1*], Elizabeth K M Johnstone[2, 3, 4*], Bincy Baby[1], Heng B See[2, 3, 4], Angela Song[5], K Johan Rosengren[5], Kevin D. G. Pfleger[2,3,4, 6], Mohammed Akli Ayoub[1,7#], and Ranjit Vijayan[1#]

1 Department of Biology, College of Science, United Arab Emirates University, PO Box 15551, Al Ain, United Arab Emirates
2 Molecular Endocrinology and Pharmacology, Harry Perkins Institute of Medical Research, QEII Medical Centre, Nedlands, WA, Australia
3 Centre for Medical Research, The University of Western Australia, Crawley, WA 6009, Australia
4 Australian Research Council Centre for Personalised Therapeutics Technologies, Australia
5 School of Biomedical Sciences, Faculty of Medicine, The University of Queensland, St Lucia, QLD 4072, Australia
6 Dimerix Limited, Nedlands, WA, Australia
7 Zayed Center for Health Sciences, United Arab Emirates University, PO Box 15551, Al Ain, United Arab Emirates

* A.A. and E.K.M.J. contributed equally to the work.

# Correspondence: M.A.A: mayoub@uaeu.ac.ae, Tel.: +971 3 713 6721; R.V.: ranjit.v@uaeu.ac.ae; Tel.: +971 3 713 6302

Supplementary Materials

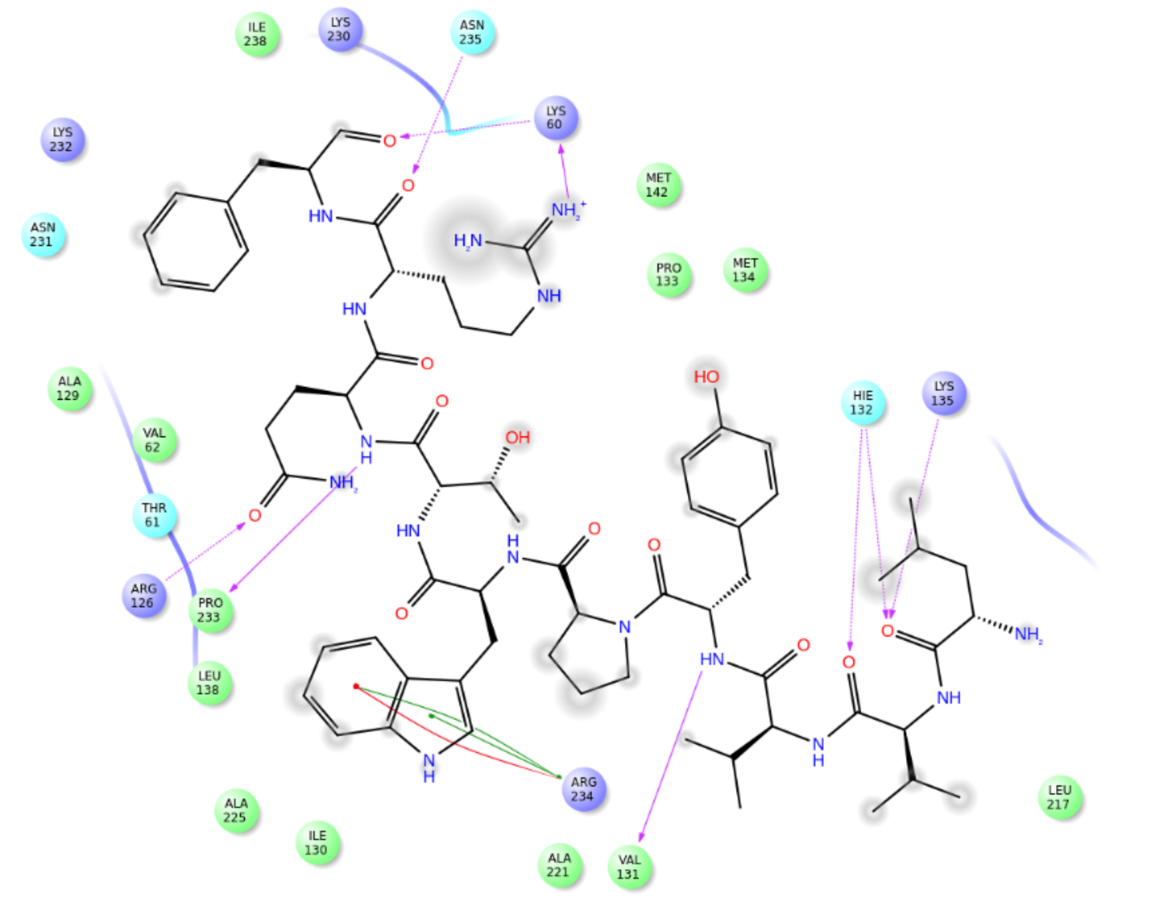

Supplementary Figure S1. 2D ligand interaction diagram of LVV-H7 bound to AT1R. AT1R (PDB ID: 4ZUD) residues that interacted with LVV-H7 in the intracellular binding site. Colored circles represent AT1R amino acids that interact with bound peptide. Red circles indicate negatively charged amino acids, dark blue colors indicate positively charged amino acids, light blue circles indicate polar amino acids, and green circles indicate hydrophobic amino. Hydrogen bonds are shown with purple arrows–dashed arrows for hydrogen bonds involving amino acid side chain and regular arrows for hydrogen bonds involving amino acid backbone. π–π interactions are represented with green lines.

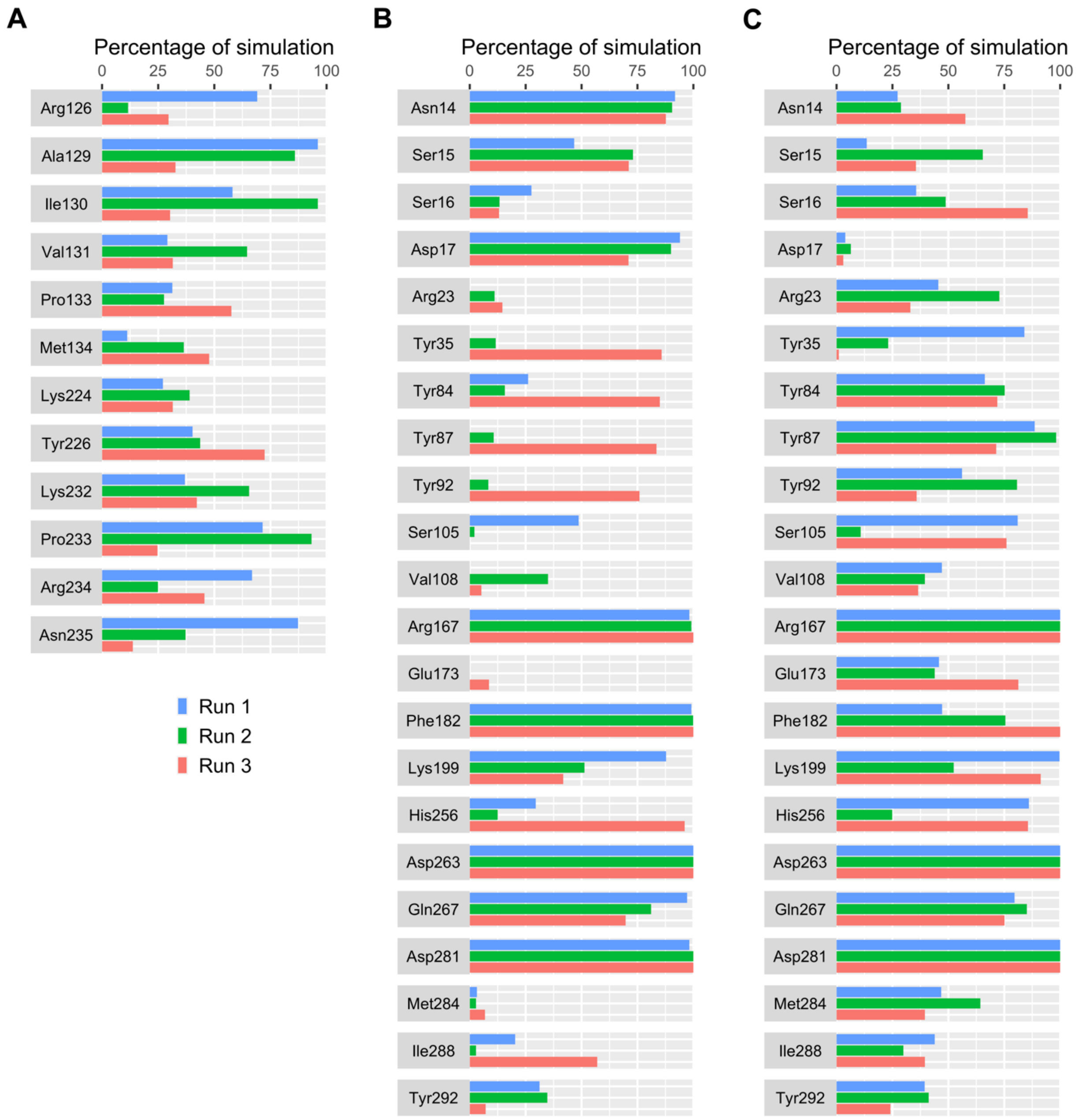

Supplementary Figure S2. Histograms of the percentage of simulation time where an AT1R residue maintained contact with: (**A**) LVV-H7 in the AT1R-LVV-H7 complex; (**B**) AngII in the AT1R-AngII complex; (**C**) AngII in the AT1R-LVV-H7-AngII complex.

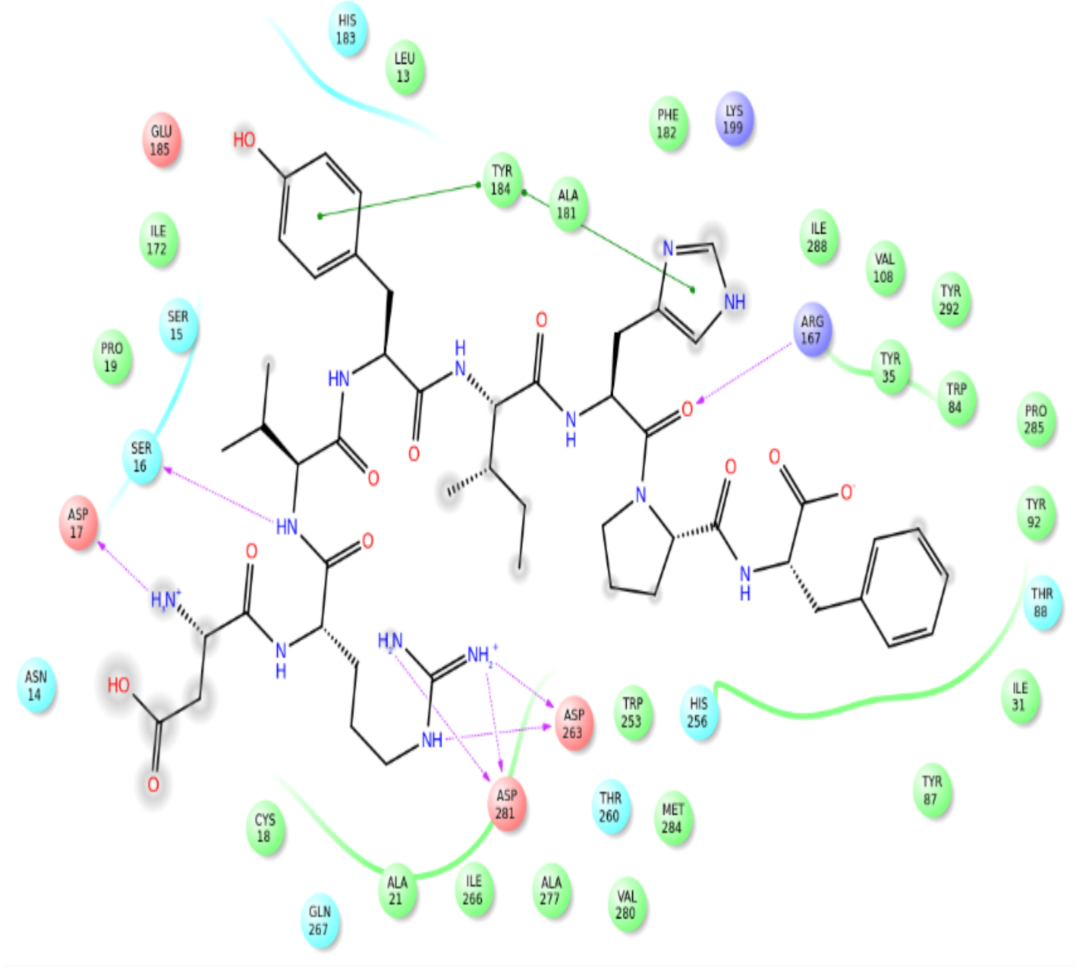

Supplementary Figure S3. 2D ligand interaction diagram of AngII bound to AT1R. AT1R (PDB ID: 4ZUD) residues that interacted with AngII in the orthosteric binding site. Colored circles represent AT1R amino acids that interact with bound peptide. Red circles indicate negatively charged amino acids, dark blue colors indicate positively charged amino acids, light blue circles indicate polar amino acids, and green circles indicate hydrophobic amino. Hydrogen bonds are shown with purple arrows–dashed arrows for hydrogen bonds involving amino acid side chain and regular arrows for hydrogen bonds involving amino acid backbone. π–π interactions are represented with green lines.

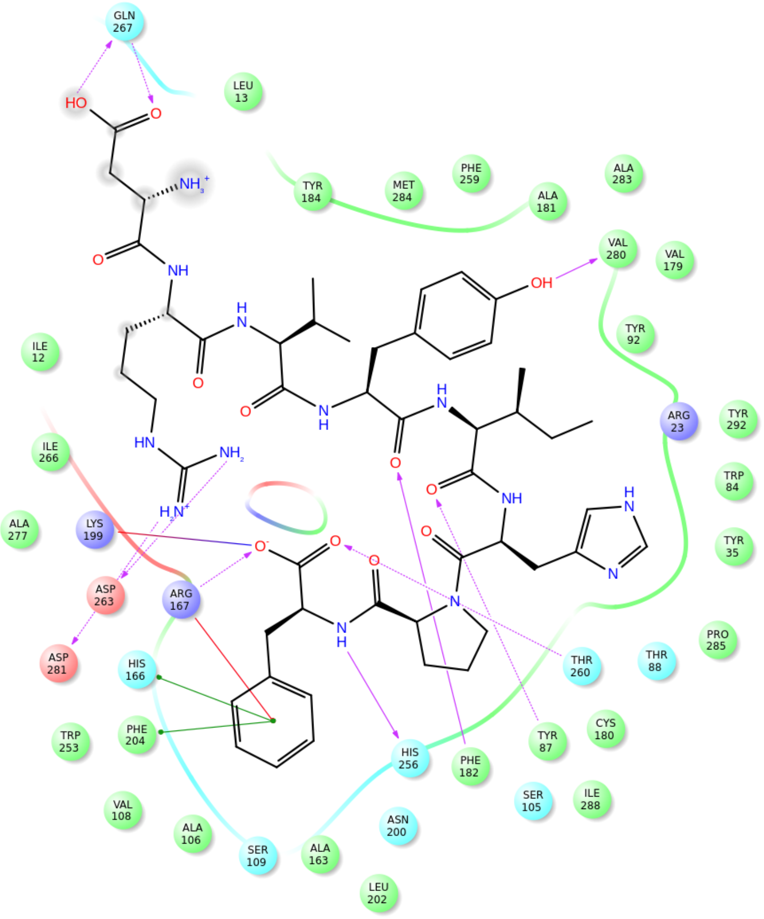

Supplementary Figure S4. 2D ligand interaction diagram of AngII bound to AT1R-LVV-H7 complex. AT1R residues that interacted with AngII in the orthosteric binding site. Colored circles represent AT1R amino acids that interact with bound peptide. Red circles indicate negatively charged amino acids, dark blue colors indicate positively charged amino acids, light blue circles indicate polar amino acids, and green circles indicate hydrophobic amino. Hydrogen bonds are shown with purple arrows–dashed arrows for hydrogen bonds involving amino acid side chain and regular arrows for hydrogen bonds involving amino acid backbone. π–π interactions are represented with green lines.
